# Impact of extent thyroidectomy and radioactive iodine ablation for disease free survival in the intermediate-risk patients with lateral neck lymph node metastasis: a retrospective and tentative real-world approach

**DOI:** 10.1186/s12957-025-03912-0

**Published:** 2025-07-03

**Authors:** Kiyomi Horiuchi, Yuki Yamanashi, Tomoyoshi Nakai, Juro Yanagida, Yusaku Yoshida, Yoko Omi, Takahiro Okamoto

**Affiliations:** Department of Endocrine Surgery, Tokyo Women’s Medical University, 8-1 Kawada-cho, Shinjuku-ku, Tokyo, 1628666 Japan

**Keywords:** Papillary thyroid cancer, Intermediate risk, Radioactive iodine ablation, Thyroidectomy

## Abstract

**Background:**

It is controversial whether or not total thyroidectomy followed by radioactive iodine ablation (RAI-a) (30 mCi = 1.1 GBq) is mandatory in intermediate-risk patients with lateral neck lymph node metastasis (cN1b).

**Methods:**

This retrospective observational study enrolled PTC patients classified preoperatively as the intermediate-risk with cN1b from January 2010 to December 2017 according to the guidelines for thyroid tumors of the Japanese Association of Endocrine Surgeons (JAES) since 2009. We analyzed recurrence-free survival (RFS) rates estimated by the Kaplan–Meier method in the patients classified into three groups: 1) total thyroidectomy (TTx) followed by lateral neck lymph node dissection (LLND) with RAI-a, 2) TTx + LLND without RAI-a, 3) hemithyroidectomy + LLND. In addition, propensity score analysis adjusted by clinical parameters was performed.

**Results:**

Two hundred eighty-eight patients with intermediate-risk PTC were identified. Among them, 87 patients with cN1b were analyzed.

Five-year RFS rates in 1), 2), and 3) groups were 85.0%, 100%, and 90.9%, respectively. The analysis of 1) and 2) groups by propensity score matching revealed 5-yr RFS rates of 84.1% and 100%, respectively (*p* < 0.0432).

**Conclusions:**

There was no evidence to support the use of 30 mCi (1.1GBq) radioactive iodine postoperatively to prevent recurrence in intermediate-risk PTC patients with cN1b.

## Introduction

Papillary thyroid cancer (PTC) is a slowly growing tumor that has a better prognosis than other histopathological types of thyroid cancer. Most PTCs do not pose a threat to the patient’s life.

In 2018, the Japan Association of Endocrine Surgeons (JAES) revised the guidelines for managing thyroid tumors. [1] According to the revised guideline, there are four recurrence risk category stratifications for PTC: very-low, low, intermediate, and high-risk. Very low-risk and low-risk PTCs include T1a (Tumor < 1.0 cm) N0 M0 and T1b (1.0 cm < Tumor ≤ 2.0 cm) N0 M0 tumors according to the Union for International Cancer Control (UICC) classification. In contrast, high-risk cancers are defined as those whose diameter is more than 4 cm, or with lymph node metastasis larger than 3 cm in diameter, or extending beyond the sternothyroid muscle, such as to the laryngeal nerve, trachea, jugular vein, or the common carotid artery. The intermediate-risk group includes tumors between 2 to 4 cm in diameter that are positive for lateral neck lymph node metastasis of less than 3 cm or with extra-thyroid extension to the muscles surrounding the thyroid. The JAES guidelines recommend surgical and post-operative treatment for PTC, with hemithyroidectomy (HTx) and central lymph node dissection (CLND) for low-risk PTC, and total thyroidectomy (TTx) and central + therapeutic lateral neck lymph node dissection (LLND) followed by radioactive iodine (RAI) for high-risk PTC. However, there is no explicit strategy for intermediate-risk PTC.

We previously reported no evidence of the usefulness of radioactive iodine ablation (RAI-a) (30 mCi = 1.1GBq) in the intermediate-risk group classified by JAES guidelines [2]. However, the usefulness of RAI-a in intermediate-risk PTC patients with lateral neck lymph node metastasis (cN1b) still needs to be clarified. Since cN1b is one of the prognostic factors predicting the recurrence of PTC, it is natural to devise the therapeutic strategy of mandatory TTx + LLND followed by RAI as ablation/adjuvant therapy for patients with intermediate-risk PTC with cN1b. In Japan, however, the use of “adjuvant” or “therapeutic” RAI, with a dose of over 100 mCi (3.7 GBq), is limited due to the lack of facilities that meet the legal requirements for administering this treatment. Hence, patients needing more than 100 mCi (3.7 GBq) RAI must wait approximately half a year, especially in large cities such as Tokyo. Therefore, a 30 mCi (1.1 GBq) dose is administered as ablation therapy, vital in treating intermediate-risk PTC in Japan. However, there is limited research evaluating the prognosis of intermediate-risk PTC patients stratified by the extent of surgical resection and administration of post-operative RAI-a. This study compared recurrence-free survival (RFS) among three surgical and post-operative strategies: TTx+ LLND with or without RAI-a and HTx+ LLND. In addition, we compared the prognosis of TTx + LLND with or without RAI-a using propensity score matching to control for confounding.

## Methods

**Fig. 1:**
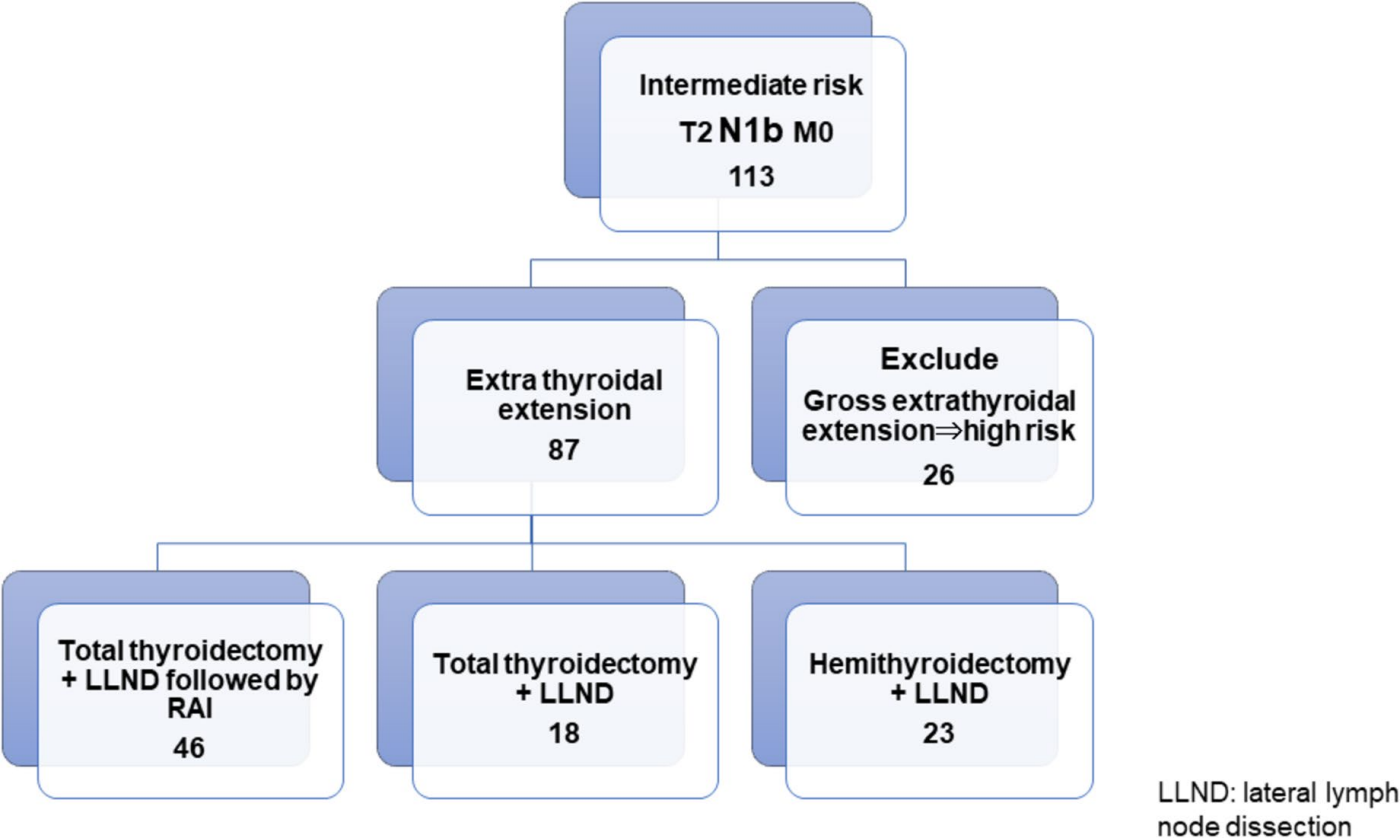
Inclusion criteria of the patients

This retrospective study analyzed PTC patients who underwent thyroid surgery at Tokyo Women’s Medical University. We obtained data on cases of PTC with cN1 diagnosed preoperatively as being of intermediate-risk according to the JAES. cN1b was diagnosed preoperatively by ultrasound examination and was confirmed by fine needle aspiration cytology. Since the JAES guidelines were revised in 2018, some patients preoperatively diagnosed with intermediate-risk PTC were retrospectively reclassified into the high-risk group in this study and excluded from the analysis. All the included patients had undergone total/hemithyroidectomy and therapeutic uni-/bi-lateral lymph node dissection. The surgical approach and the decision on whether to administer additional RAI are basically determined by the outpatient physician. For intermediate-risk patients, our standard procedure is hemithyroidectomy. Total thyroidectomy is performed in case of multifocal disease. Whether or not to administer RAI after total thyroidectomy is also at the discretion of the outpatient physician. From 2010 to 2017, 288 patients were diagnosed with intermediate-risk PTC. Of these, 113 patients had cN1b, among whom 26 patients were excluded from the study because of massive extrathyroidal extension beyond the sternothyroid muscle, based on which they were reclassified into the high-risk group. (Fig. 1).

We divided the remaining 87 patients into three groups according to the extent of thyroidectomy and postsurgical treatment 1) TTx+ LLND followed by RAI-a, 2) TTx+ LLND, and 3) HTx+ LLND (Fig. 1). RAI-a was defined as ablation using 30 mCi (1.1GBq) RAI postoperatively, followed by a diagnostic 10 mCi (0.37GBq) whole-body scan after six months. No accumulation on whole-body scanning indicated the success of RAI-a. In case of remnant accumulation, one-time 100 mCi (3.7GBq) therapy or repeated 30 mCi (1.1 GBq) RAI doses were administered until the disappearance of the accumulation or if decided by the radiologists. [3] The Response initial treatment was evaluated in accordance with the American Thyroid Association (ATA) guidelines.

The primary outcome of this study was a comparison of RFS rates between the three surgical strategy groups. The secondary outcome was evaluating RFS rates between the two TTx groups adjusted for sex, age, tumor size, and number of lateral neck lymph node metastases by propensity score matching. RFS was determined based on the identification of metastasis by structural examination, such as US or CT. Thyroglobulin (Tg) can serve as a useful tumor marker, particularly in the detection of distant metastasis. However, its accuracy may be compromised in the presence of anti-thyroglobulin antibodies (TgAb), which interfere with Tg measurement. Notably, serum Tg becomes a more sensitive tumor marker after radioactive iodine ablation (RAI-a) compared to cases without RAI-a. Therefore, in this study- which included three treatment modalities: hemithyroidectomy, total thyroidectomy alone, and total thyroidectomy followed by RAI-a—an isolated increase in Tg levels without structural evidence of disease was not considered indicative of recurrence. When assessing RFS, the day of surgery was considered day 0, and the day of the last visit to the outpatient clinic was considered the day until recurrence. The Kruskal–Wallis test was used to compare continuous variables among the three groups, and RFS was calculated using the Kaplan–Meier method and compared using the log-rank test. Statistical significance was set at *p*<0.05. For multivariable analysis using a Cox proportional hazard model, we considered sex, age (55 years old), tumor diameter (2 cm), extrathyroidal extension, thyroidectomy, post-operative RAI-a, and post-operative TSH suppression as confounding factors.

In general, TTx followed by RAI-a is preferable for patients with more aggressive cancers, such as a large tumor or multiple LN metastasis. Since several confounders sometimes prevent accurate analysis, we attempted to adjust for all confounders in this study. We assumed that sex, age, tumor diameters, and the number of lateral neck lymph node metastases would influence the choice of RAI-a. Hence, we conducted a propensity score (PS) matching analysis between the TTx + LLND group and the TTx + LLND followed by the RAI-a group, with a PS model estimated using a logistic regression model that adjusted for the above confounders. One-to-one pair matching of a “TTx + LLND followed by RAI-a” patient with a “TTx + LLND” patient was performed using nearest-neighbor matching without replacement. The caliper was estimated to be 0.2 of the standard deviation of the logit of the propensity score.

The institutional review board of Tokyo Women’s Medical University approved this clinical study (# 2021–0174), and it was conducted in accordance with the principles of the Declaration of Helsinki.

## Results

### Patient’s background characteristics

The 87 patients evaluated included 28 males and 59 females, ranging in age from 15–78 years (median: 51 years). The median follow-up period was 38.7 months, ranging from 1 to 101 months. Eight patients experienced recurrence, although there were no cause-specific deaths. Table 1 shows the demographic and pathological information of the patients and the three groups. In two patients who were diagnosed with lateral lymph node metastasis by fine needle aspiration biopsy, post-operative histopathological diagnosis revealed no lymph node metastasis. There were no statistically significant differences in serum Tg levels or in the number of patients with positive TgAb among the three groups (*P* = 0.232, *P* = 0.818) Similarly, the ratios of lateral lymph node metastasis did not differ significantly among the groups (*P* = 0.510). No RAI scans revealed any evidence of distant metastasis. The extent of surgery and RAI-a increased by cN1b. The Kruskal–Wallis test revealed a statistically significant difference in the number of lateral lymph node metastases between the three groups, specifically between the TTx followed by RAI-a group and the HTx group (*P* = 0.031).

**Table 1:** Patients’ background characteristics

	Total	TTx + LLND followed RAI-a	TTx + LLND	HTx + LLND	*P*-value
Patients number	87	46	18	23	
Male/Female	28/59	17/29	4/14	7/16	0.560
Age	51 (15 ~ 79)	52 (27 ~ 78)	47.5 (15 ~ 74)	46 (21 ~ 79)	0.107
Follow up periods (months)	38.7 (1 ~ 101)	39.3 (9 ~ 81.8)	58.2 (1 ~ 101.1)	24.1 (11.3 ~ 92.7)	0.193
Tg antibody					0.818
Positive	14	6	3	5	
Negative	73	40	15	18	
Preoperative Tg	42.8 (0.3 ~ 438.8)	32.25 (0.7 ~ 377.1)	59.85 (0.3 ~ 280.2)	45.90 (0.9 ~ 438.8)	0.232
Tumor diameters (mm)	17.8 (1.8 ~ 40)	18.5 (4 ~ 39)	18.5 (1 ~ 38)	16 (6 ~ 40)	0.469
Extra thyroid extension	47	27	9	11	0.627
Multifocal	37	26	6	6	0.073
Number of lateral	2 (0 ~ 13)	3 (0 ~ 13) *	2.5 (1 ~ 8)	2 (0 ~ 8) *	0.031*
LN metastasis					
Ratio of lateral neck LN metastasis	0.22 (0 ~ 1)	0.25 (0 ~ 1)	0.27 (0.04 ~ 1)	0.17 (0 ~ 0.67)	0.510
Response of initial treatment					4.9* × 10^–5^*
Excellent	27	22	5	0	
Biochemical Incomplete	26	16	3	7	
Indeterminate	34	8	10	16	
Tg at the endpoint(median)	0.7	0.3	0.4	10.6	1.19 × 10^–7^*
Recurrence	8	7	0	1	
Death	1	0	1	0	

*TTx* total thyroidectomy, *LLND* lateral neck lymph node dissection median (range), *Tg* Thyrogloburin

*P < 0.05

### Unadjusted outcomes

**Fig. 2:**
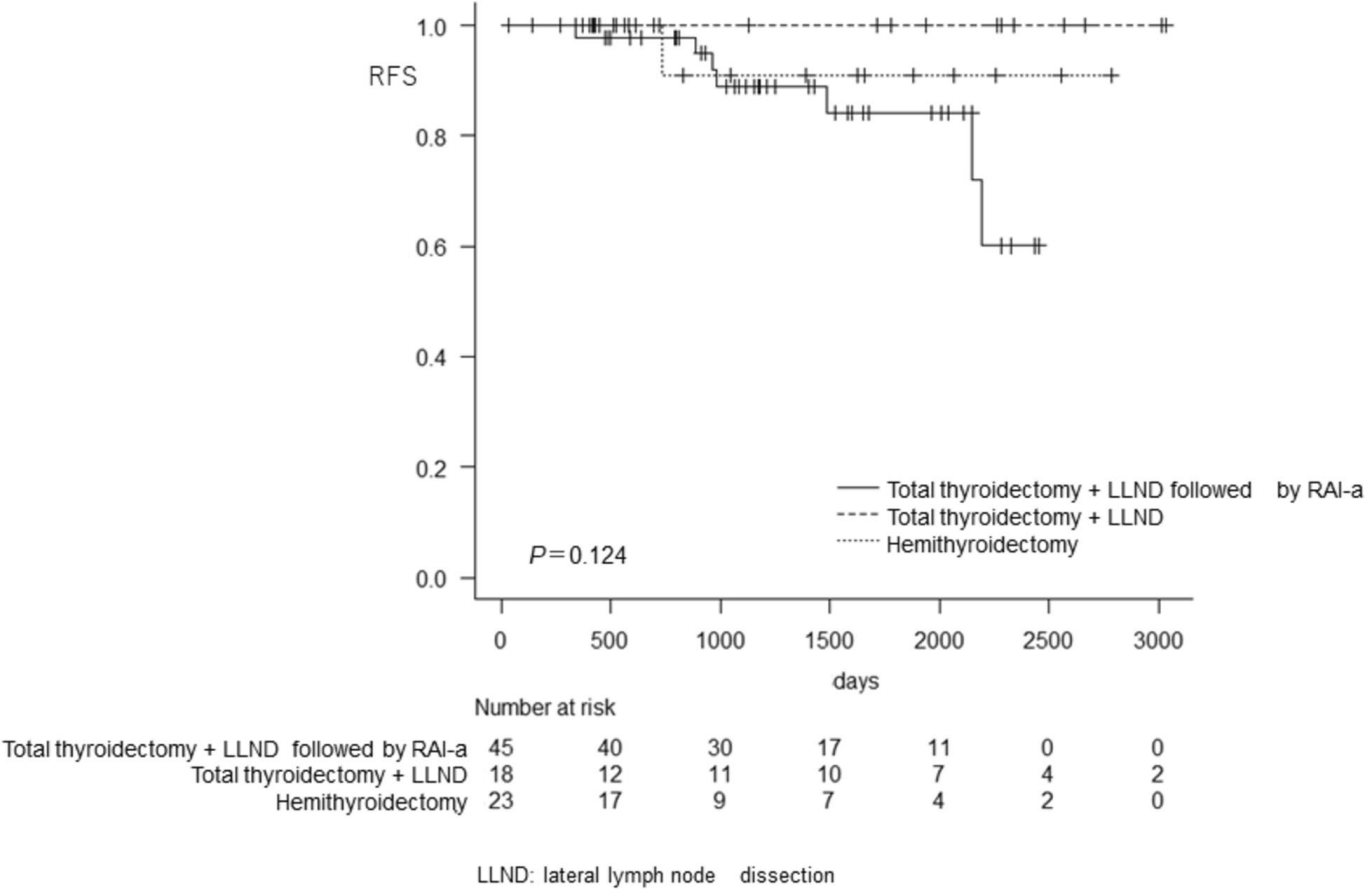
Recurrence-free survival in the three groups

Figure 2 shows RFS rates in the three groups. The total thyroidectomy group had a better RFS rate than the other two. The 8-year RFS rates (95% confidence interval) in the HTx and TTx followed by RAI-a groups were 90.9% (86.7‒98.9) and 84.1% (64.5‒93.4), respectively. On the other hand, RFS was 100% at eight years following TTx without RAI. However, there were no statistically significant differences between the three groups (*P*=0.124). Multivariable analysis did not reveal any factors associated with the risk of RFS (Table 2).

**Table 2:** Multivariable analysis using a Cox proportional hazards model

	HR	95% CI	*P*-value
Age > 55	1.744e + 00	0.428 ~ 7.100	0.437
Diameter > 2 cm	1.151e + 00	0.254 ~ 5.199	0.854
Gross Ex	3.353e-01	0.038 ~ 2.884	0.319
RAI-a	6.362e + 08	0.000 ~ Inf	0.998
Sex M	1.544e-01	0.018 ~ 1.315	0.087
Thyroidectomy	6.804e-09	0.000 ~ Inf	0.998
TSH suppression	5.372e-01	0.140 ~ 2.048	0.362

*Ex* extrathyroidal extension, *RAI-a* radio-active iodine ablation

### Propensity score matching

Multivariable regression of propensity for RAI-a had an area under the receiver operating characteristics curve (ROC) of 0.659 (95% confidence interval (CI): 0.493‒0.826). The total thyroidectomy followed by RAI-a group had a mean propensity score of 0.746 (95% CI: 0.71‒0.782), while the total thyroidectomy alone group had a mean propensity score of 0.635 (95% CI: 0.534‒0.736). The balance between the groups was assessed by calculating standardized mean differences. Tables 3 and 4 show *P*-values and standardized mean differences of each variable, and the average of the standardized mean differences before and after propensity score matching were 0.306 (95% CI: 0.011‒0.601) and 0.405 (95% CI: −0.133‒0.94), respectively, in the TTx+ LLND with and without RAI-a groups.

**Table 3:** Unadjusted *p*-values and standardized differences between total thyroidectomy + LLND followed by RAI-a versus total thyroidectomy + LLND alone groups

	TTx + LLND followed RAI-a	TTx + LLND	*P*-value	Standardized mean differences
Number	46	18		
Sex				
Male	17	4	0.405	0.327
Female	29	14		
Age	52.5	44.4	0.036*	0.549
Number of lateral				
LN metastasis	5.54	4.61	0.418	0.240
Tumor diameter	19.04	20.07	0.694	0.108

*TTx* total thyroidectomy, *LLND* lateral lymph node metastasis, *LN* lymph node

^*^P < 0.05

**Table 4:** Adjusted *p*-values and standardized differences between total thyroidectomy + LLND followed by RAI-a versus total thyroidectomy + LLND alone groups after propensity score matching

	TTx + LLND followed RAI-a	TTx + LLND	*P*-value	Standardized mean differences
Number	14	14		
Sex				
Male	0	4	0.105	0.894
Female	14	10		
Age	51.4	50.3	0.820	0.087
Number of LN metastasis	5.29	4.57	0.591	0.205
Tumor diameter	15.49	19.27	0.217	0.478

*TTx* total thyroidectomy, *LLND* lateral neck lymph node dissection, *LN* lymph node

### Adjusted outcomes

**Fig. 3:**
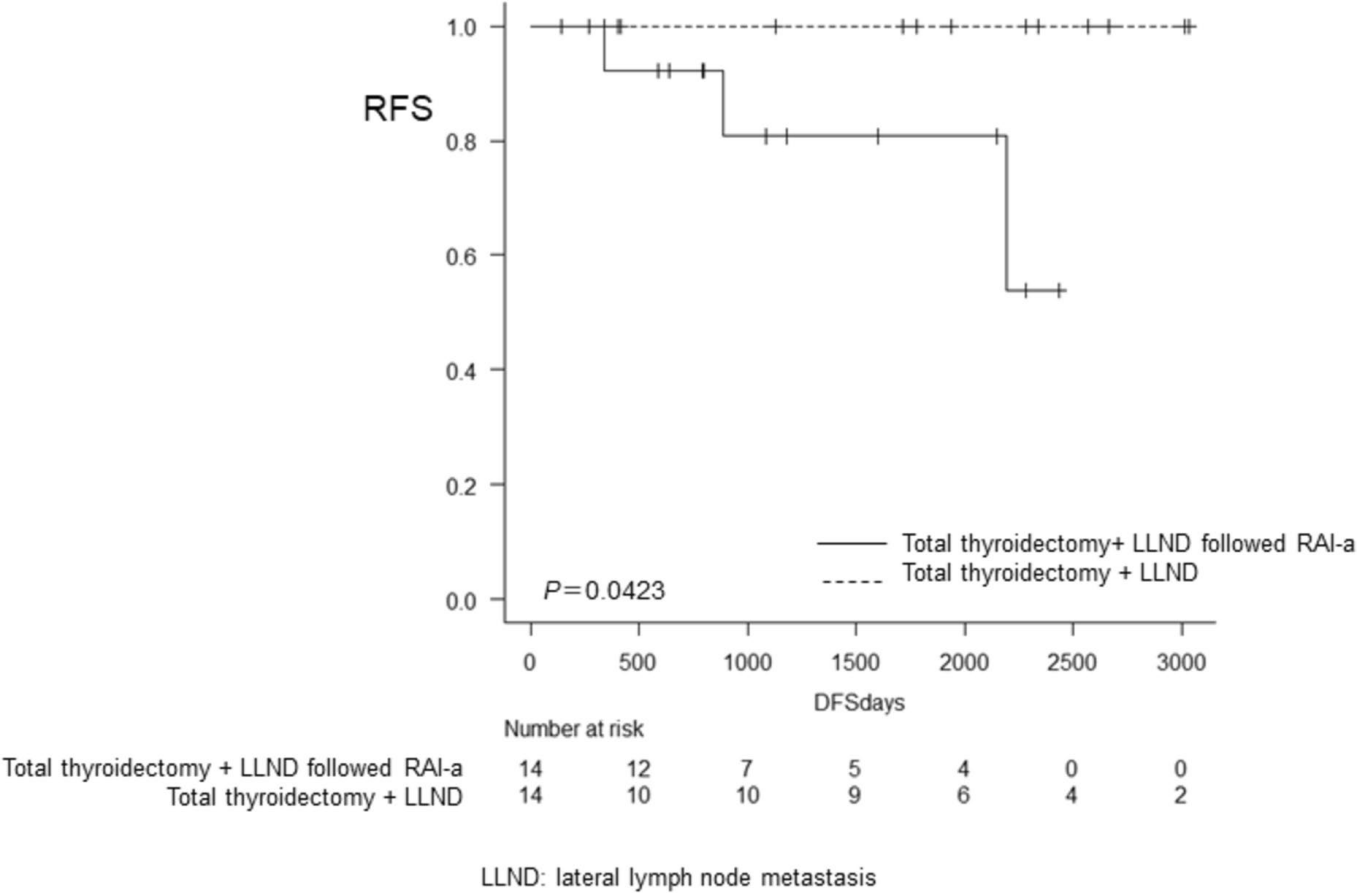
Recurrence-free survival with total thyroidectomy + LLND followed by RAI vs. total thyroidectomy + LLND alone using propensity score matching

Figure 3 shows the RFS of the TTx group and the TTx followed by the RAI-a group after propensity score matching. RFS rates of the TTx + LLND followed by RAI-a group at 3, 5 and 8 years were 89.0% (95% CI; 73.1‒95.8), 84.1% (95% CI; 64.5‒93.3), and 60.6% (95% CI; 26.4‒82.4) respectively. The RFS rate of the TTx + LLND alone group was 100% at eight years, with a statistically significant difference between the two groups (*P*=0.0432).

## Discussion

This report is the first to evaluate the effect of the extent of thyroidectomy and administration of post-operative RAI-a on intermediate-risk PTC with lateral neck lymph node metastasis classified according to JAES guidelines. Our study revealed that the RFS rates of TTx alone and HTx were better than those of TTx followed by RAI-a. The uniqueness of our study was that we used propensity score matching to compare RFS between the TTx + LLND group and TTx + LLND followed by the RAI-a group to adjust for confounders.

We previously reported that RAI-a is ineffective in preventing recurrence in the intermediate-risk group of PTC. However, RAI-a would be effective for the particular group. Several studies have reported that lateral neck lymph node metastasis is one of the poor prognostic factors in PTC. [4–6] Some studies have focused on the number of positive lateral neck lymph nodes. Dong et al. revealed that the ratio of positive lymph nodes/total number of lymph nodes strongly correlates with a poor prognosis, with a worse prognosis with a ratio > 0.5. [7]

The extent of thyroidectomy required for the intermediate-risk group of PTC is controversial. 2007 Bilimoria et al. analyzed disease-specific survival (DSS) and overall survival (OS) using the National Cancer Database from 1995 to 1998. They revealed that a tumor diameter of more than 1 cm was statistically significantly associated with the risk after lobectomy, with a 15% higher risk of recurrence and about 30% higher risk of death. [8] Wang et al. compared RFS following TTx and HTx in patients with T2N1b. In their results, the 5-year RFS rates following TTx and HTx were 97.7% and 97.4%, respectively, [9] indicating no statistically significant difference. Our results were similar to these previous results. Adams et al. also analyzed the OS of PTC patients with tumors ranging from 1 to 4 cm from the national cancer database. They pointed out that TTx patients had more nodal/extrathyroidal tumor extension and multifocal disease. By adjusting for the patient’s demographic and clinical factors, the OS of both TTx and HTx was similar, with no statistical significance (*p* = 0.54). [10] In contrast, Rajjoub et al. reported that the OS of TTx was better than that of HTx in patients with T2 disease. [11] However, the limitation of their study was the lack of precise information on lateral neck lymph node metastasis.

Decision-making regarding the extent of surgery and conduct of RAI depends on the severity of the cancer. Comparison of the prognosis between RAI-a and non-RAI groups is likely to be affected by confounding patient characteristics. The three strategies for coping with confounders and resolving these issues are stratification, statistical adjustment, and propensity score matching. [12] In our study, we chose propensity score analysis to overcome confounding. Propensity scores are helpful in observational studies related to treatment efficacy for controlling confounding by indication. Our study’s adjusted analysis by propensity score matching showed that after TTx, there was no benefit of RAI-a in patients with intermediate-risk PTC.

Generally, it is natural to consider that RAI therapy with a 100 mCi dose (distinct from ablation) might be adequate to prevent recurrence in intermediate-risk PTC patients. [13] However, it is unclear whether 30 mCi ablation is a sufficient dose for preventing recurrence. Furthermore, an interesting paper was recently published on papillary thyroid microcarcinoma (PTMC) with palpable lateral neck lymphadenopathy. [14] In that study, the DFS of the PTMC with palpable lateral neck lymphadenopathy group, who underwent TTx followed by adjuvant RAI, was inferior to that of the PTMC with N0 group, among whom only 48% had RAI. Hence, whether or not RAI-a (30 mCi=1.1GBq)) should be administered to PTC patients in the intermediate-risk group with lateral neck lymph node metastasis is still controversial. [2]

Our study could not prove any benefit in terms of RAI-a after TTx in patients with intermediate-risk PTC. It is unclear whether RAI ablation therapy (30 mCi = 1.1 GBq) itself may cause recurrence of PTC or not. RAI ablation may cause activation of specific molecular pathways related to thyroid cancer recurrence, we could not find any previous reports describing a relationship between RAI and molecular pathway activation of PTC. Theoretical mechanisms linking RAI ablation to molecular pathway activation remain unexplored. We speculate that ablation may enhance the sensitivity to Tg elevation, potentially leading to earlier imaging studies and facilitating the detection of recurrence.

There are some limitations to this study. First, the number of patients was small. After propensity score matching, there were only 28 patients (14 vs. 14). This limited sample size may lead to insufficient statistical power and potential false-positive results. Second, some subjects had a short follow-up period of less than eight years, which may lead to potential selection bias. Third, we evaluated the efficacy of RAI-a (radiation dose: 30 mCi = 1.1 GBq) and not RAI therapy, such as more than 100 mCi (3.7GBq). Hence, we cannot comment on the efficacy of a high dose of RAI therapy of ≥ 100 mCi(3.7GBq).

Further, in this study, the outcome was RFS and not OS. Even though our study revealed that there was a relationship between RAI-a and post-operative recurrence, RAI-a may not influence OS in patients with intermediate-risk PTC. The last limitation is the propensity score matching. The AUC of the ROC of the propensity score was less than 0.8 which means the model’s goodness (multivariable analysis) of fit was not preferrable enough, and the adjusted standardized differences were not less than 0.1, indicating that the balance of the covariates was insufficient. This insufficient balance might have been due to the small number of patients.

## Conclusions

Our study implies that postoperatively administered RAI at a dose of 30 mCi (1.1GBq) for tumor ablation might not be adequate for preventing recurrence in patients with intermediate-risk PTC with cN1b. Hence, the utility of the administration of 30 mCi (1.1GBq) of RAI as ablation therapy in intermediate-risk PTC patients with cN1b is still controversial.

## Data Availability

No datasets were generated or analysed during the current study.

## Abbreviations

JAES: Japanese Association of Endocrine Surgeons
RFS: Recurrence-free survival
TTx: Total thyroidectomy
LLND: Lateral neck lymph node dissection
PTC: Papillary thyroid cancer
HTx: Hemithyroidectomy
CLND: Central lymph node dissection
PS: Propensity score
ROC: Receiver operating characteristics curve
CI: Confidence interval
DSS: Disease-specific survival
OS: Overall survival
PTMC: Papillary thyroid microcarcinoma
